# Enhanced COVID-19 Provider Relief, Hospital Finances, and Care for Medicare Inpatients

**DOI:** 10.1001/jamahealthforum.2025.0046

**Published:** 2025-03-07

**Authors:** Jason D. Buxbaum

**Affiliations:** 1 Department of Health Care Policy, Harvard Medical School, Boston, Massachusetts; 2 Now with Center for Advancing Health Policy through Research, Department of Health Services, Policy, and Practice, Brown University School of Health, Providence, Rhode Island

## Abstract

**Importance:**

Congress appropriated $178 billion in emergency relief for health care providers (hospitals, physicians, and other health care professionals) in 2020 to stabilize finances and support the COVID-19 pandemic response. The US Department of Health and Human Services directed $35 billion of these funds to safety-net hospitals and high-impact hospitals using strict criteria. However, the importance of enhanced funding is inadequately understood.

**Objective:**

To evaluate the association between enhanced COVID-19 relief funding and hospital finances and clinical care for Medicare inpatients.

**Design, Setting, and Participants:**

This retrospective cohort study used a differences-in-discontinuities study design with overlap weighting. Nonrural hospitals with data on costs and fee-for-service Medicare inpatient care for 2018 to 2021. Hospitals near a threshold for receiving high-impact and/or safety-net hospital funding were analyzed. Data were analyzed from July 2022 to January 2025.

**Exposures:**

Receipt of high-impact and/or safety-net hospital relief funds.

**Main Outcomes and Measures:**

Financial outcomes related to revenues, costs, margin, and liquidity and clinical outcomes related to volume, care processes, and mortality.

**Results:**

A total of 555 hospitals were included, with 311 receiving high-impact and/or safety-net hospital funds. Hospitals not receiving enhanced relief averaged $7.0 million in total relief (about $45 000 per bed), while hospitals receiving enhanced relief averaged $15.4 million in total relief (about $100 000 per bed). Operating revenues in 2020 increased by 4.5% (95% CI, 3.0-5.9) among basic relief hospitals and 6.1% (95% CI, 4.6-7.6) among enhanced relief hospitals. However, total costs grew similarly (basic relief: 4.6%; 95% CI, 3.6-5.6; enhanced relief: 4.5%; 95% CI, 3.4-5.7). This resulted in a significant differential increase of 1.4 points (95% CI, 0.3-2.5) in operating margin in association with enhanced relief. Enhanced relief was also associated with limited deterioration in liquidity (differential increase in net asset ratio of 0.03 points; 95% CI, 0-0.05). There was not a significant association between receipt of enhanced relief and fee-for-service Medicare inpatient admissions (−19.6 stays; 95% CI, −281.0 to 241.8), use of a sentinel deferrable procedure among fee-for-service Medicare inpatients (−3.9 admissions for lower joint replacement; 95% CI, −29.6 to 21.7), or use of 2 resource-intensive services among fee-for-service Medicare inpatients (−0.3 admissions with ventilation; 95% CI, −20.8 to 20.2; 0.9 admissions with dialysis; 95% CI, −15.4 to 17.1). Enhanced relief was not detectibly associated with change in the complexity (change in Charlson Comorbidity Index score, 0 points; 95% CI, 0-0) or inpatient mortality (−2.9 deaths; 95% CI, −11.3 to 5.5) for fee-for-service Medicare inpatients.

**Conclusions and Relevance:**

In this study, enhanced emergency relief for hospitals was associated with improved margins and liquidity without detectible changes in spending or service provision.

## Key Points

**Question:**

COVID-19 pandemic relief funds stabilized hospital finances, but what is the association between funding generosity and changes in hospital finances and operations?

**Findings:**

In this cohort study of 555 hospitals, receipt of enhanced relief was associated with increased margins and improved liquidity but no detectible changes in expenditures or service delivery.

**Meaning:**

Adjusted approaches to relief distribution may maximize the impact of funding on clinical care and hospital operations during emergencies without differentially improving finances among similarly situated hospitals.

## Teaser

This cohort study evaluates the association between enhanced COVID-19 relief funding and hospital finances and clinical care for Medicare inpatients.

## Introduction

Waves of COVID-19 overwhelmed the US’s acute care system in 2020 and 2021. Hospitals placed beds in chapels, gift shops, and parking garages as ambulances idled outside overflowing emergency departments.^1,2^ The *Chicago Tribune* described one hospital as “outgunned, outmanned, and underfunded”^3^; the *New York Times* reported on another overwhelmed by an “‘apocalyptic’ Coronavirus surge.”^4^ Differences in early COVID-19 pandemic resources likely contributed to a 12-fold variation in postdischarge survival across hospitals.^5,6^ At the same time, overall inpatient volume decreased outside local disease surges, and costs for supplies and personnel spiked. Hospital advocates warned of difficulty making payroll,^7^ and experts warned of bankruptcies, closures, and consolidation.^8^

The March 2020 Coronavirus Aid, Relief, and Economic Security Act supplied a first tranche of $100 billion to support health care providers (hospitals, physicians, and other health care professionals) in “prevent[ing], prepar[ing] for, and respond[ing] to coronavirus . . . [and to reimburse] eligible health care providers for health care related expenses or lost revenues that are attributable to coronavirus.”^9^ Additional legislation brought the total to $178 billion.^10,11^ Within these broad parameters, statutory language gave broad discretion to the US Department of Health and Human Services (HHS) to allocate funding. HHS ultimately directed about one-half of all relief to hospitals.

Nearly every US hospital received 2% of historical net revenues. However, HHS used strict cutoffs when supplementing these funds, with an additional $21 billion and $13 billion for high-impact (HI) hospitals and safety-net hospitals (SNHs), respectively.^12^ HI funds were awarded to about 950 hospitals exceeding any of 3 administratively determined thresholds for COVID-19 case intensity (Table 1; eAppendix 1 in Supplement 1).^12^ SNH funds were awarded to about 900 hospitals with historically low profitability, high uncompensated care, and a high share of hospitalizations for disabled and dually eligible Medicare beneficiaries (Table 1; eAppendix 1 in Supplement 1).^12^ On average, hospitals qualifying for HI funding, SNH funding, or both (termed *enhanced relief hospitals*) received about $100 000 per bed—roughly twice the relief received by other hospitals (termed *basic relief hospitals*) (Table 2; eAppendix 2 in Supplement 1).

**Table 1.** Criteria for Receipt of Targeted COVID-19 Provider Relief Funding^a^

Funding stream and running variable	Source
Safety-net funding, meet all criteria	
Uncompensated care per bed ≥$25 000	Most recent cost report as of May 27, 2020
Disproportionate patient percentage ≥20.2%	Most recent cost report as of May 27, 2020
Profit <3% in most recent year or mean profit <3% over 2 consecutive y in last 5 y	Most recent 5 cost reports as of May 27, 2020
High-impact funding, meet any criterion	
COVID-19 cases ≥100 through April 10, 2020	Hospital-submitted case counts
COVID-19 cases per bed ≥0.54864 through June 10, 2020	Hospital-submitted counts of cases and beds
COVID-19 cases ≥161 through June 10, 2020	Hospital-submitted case counts

^a^ Hospital-reported case counts used to determine eligibility for high-impact funding were obtained from the Health Resources and Services Administration through a Freedom of Information Act request. Disproportionate patient percentage is the sum of (1) the percentage of inpatient days attributable to patients with Medicaid coverage but not Medicare Part A, and (2) the percentage of inpatient days for patients with Medicare Part A receiving supplemental security income.

**Table 2.** Characteristics of Study Hospitals Across Enhanced Funding^a^

Characteristic	Mean (SD)					
	Before weighting			After weighting		
	Basic funding (n = 244)	Enhanced funding (n = 311)	SMD	Basic funding (n = 244)	Enhanced funding (n = 311)	SMD
**Running variables**						
Safety-net hospital funding						
Lowest total margin, %	−0.1 (12.3)	−4.1 (14.4)	0.30^b^	−0.3 (12.3)	−3.2 (13.5)	0.23^c^
Uncompensated care per bed, $ in thousands	42.8 (33.3)	58.7 (55.5)	0.35^c^	46.5 (36.8)	52.3 (42.7)	0.15
Disproportionate patient percentage	31.5 (18.5)	34.6 (16.7)	0.18^d^	31.7 (18.6)	32.8 (15.7)	0.07
High-impact funding						
COVID-19 admissions through April 10, 2020	13.3 (13.3)	29.5 (26.7)	0.77^c^	14.4 (13.6)	27.5 (26.2)	0.63^c^
COVID-19 admissions through June 10, 2020	40.3 (33.6)	93.0 (70.0)	0.96^c^	44.6 (35.1)	81.2 (66.6)	0.69^c^
COVID-19 admissions per bed through June 10, 2020	0.23 (0.14)	0.47 (0.29)	1.06^c^	0.25 (0.14)	0.45 (0.28)	0.90^c^
**Relief funding^e^**						
HI relief, $ in millions	0	3.7 (4.3)	1.20^c^	0	3.1 (4.0)	1.09^c^
All relief, $ in millions	7.1 (6.4)	15.4 (18.7)	0.60^c^	7.3 (6.6)	12.6 (13.6)	0.49^c^
Relief per bed, $ in thousands	44.6 (38.7)	100.7 (70.6)	0.98^c^	45.4 (40.8)	95.9 (70.8)	0.88^c^
**General characteristics**						
Region, %^f^						
Midwest	22.5	20.9	0.21	22.9	22.9	<0.01
Northeast	13.5	9.3		11.7	11.7	
South	32.4	41.5		35.5	35.5	
West	31.6	28.3		29.9	29.9	
For-profit control, %^f^	25.8	19.9	0.14	22.0	22.0	<0.01
Large hospital system, %^f^	52.5	47.6	0.10	50.9	50.9	<0.01
Teaching status, %^f^						
Nonteaching	14.3	21.1	0.19	15.1	15.1	<0.01
Minor teaching	17.2	17.9		18.1	18.1	
Major teaching	68.4	61.0		66.8	66.8	
Fiscal year, %^f^						
Quarter 1	41.0	42.1	0.16	43.3	43.3	<0.01
Quarter 2	6.6	3.5		4.8	4.8	
Quarter 3	38.1	37.0		35.9	35.9	
Quarter 4	14.3	17.4		16.0	16.0	
Capacity						
Beds^f^^,^^g^^,^^h^	176.2 (104.4)	199.3 (114.8)	0.21^d^	182.8 (107.4)	182.8 (109.5)	<0.01
Any ICU services, %^f^	90.2	94.9	0.18	93.9	93.9	<0.01
Employees, FTEs^f^^,^^g^^,^^h^	970.6 (703.2)	1171.7 (819.1)	0.26^b^	1041 (745)	1041 (767)	<0.01
Overall utilization						
All inpatient days^f^^,^^g^^,^^h^	37.0 (28.8)	44.5 (32.6)	0.24^b^	39.7 (30.2)	39.7 (31.0)	<0.01
Share of commercial/nonpublic days^f^^,^^g^	27.0 (8.8)	26.6 (8.7)	0.04	27.0 (8.9)	27.0 (8.7)	<0.01
Discharge equivalents^f^^,^^g^^,^^h^	13 762 (9008)	15 958 (9895)	0.23^b^	14 611 (9208)	14 611 (9419)	<0.01
Interhospital transfer out among FFS Medicare patients^f^^,^^g^	51.0 (31.6)	54.5 (40.5)	0.10	53.2 (31.8)	53.2 (39.7)	<0.01
Inpatient services						
Lower joint replacement among FFS Medicare patients^f^^,^^g^	148.8 (167.1)	155.9 (176.2)	0.04	149.6 (165.1)	149.6 (171.8)	<0.01
Pneumonia/influenza stays among FFS Medicare patients^f^^,^^g^	1177 (953)	1437 (1164)	0.24^b^	1276 (982)	1276 (988)	<0.01
Mechanical ventilation stays among FFS Medicare patients^f^^,^^g^	172.3 (177.2)	219.8 (217.2)	0.24^b^	184.2 (176.9)	184.2 (172.6)	<0.01
Kidney replacement therapy among FFS Medicare patients^f^^,^^g^	120.9 (123.0)	184.5 (193.2)	0.39^c^	137.9 (131.7)	137.9 (141.4)	<0.01
Income						
Operating revenues, $ in millions^f^^,^^g^^,^^h^	223.4 (171.2)	261.2 (195.1)	0.21^d^	235.9 (178.5)	235.9 (184.1)	<0.01
Total costs, $ in millions^f^^,^^g^^,^^h^	209.5 (154.5)	248.7 (179.3)	0.24^b^	222.0 (163.4)	222.0 (168.7)	<0.01
Workforce costs, $ in millions^f^^,^^g^^,^^h^	87.2 (66.9)	106.6 (79.1)	0.27^b^	93.8 (70.1)	93.8 (73.2)	<0.01
Administrative costs, $ in millions^f^^,^^g^^,^^h^	37.0 (26.7)	44.2 (31.2)	0.25^b^	39.4 (28.6)	39.4 (29.2)	<0.01
Operating margin, %^f^^,^^g^^,^^h^	3.8 (7.7)	1.2 (7.6)	0.32^c^	3.0 (7.2)	3.0 (7.2)	<0.01
Balance sheet						
Current assets, $ in millions^f^^,^^g^^,^^h^	74.2 (73.9)	82.6 (79.2)	0.11	76.0 (76.6)	76.0 (75.5)	<0.01
Current liabilities, $ in millions^f^^,^^g^^,^^h^	36.3 (37.6)	42.5 (40.3)	0.16	37.4 (36.5)	37.4 (37.0)	<0.01
Liquidity^f^^,^^g^	0.15 (0.16)	0.14 (0.18)	0.05	0.15 (0.15)	0.15 (0.18)	<0.01
Cash or cash equivalents, $ per bed^f^^,^^g^	76.5 (123.2)	62.3 (109.0)	0.12	71.2 (109.2)	71.2 (114.9)	<0.01
Population served and mortality^i^						
Black race among FFS Medicare patients, %^f^^,^^g^	8.1 (8.4)	13.5 (15.6)	0.43^c^	9.3 (9.2)	9.3 (10.8)	<0.01
Hispanic ethnicity among FFS Medicare patients, %^f^^,^^g^	2.9 (5.2)	3.8 (7.0)	0.15	3.1 (5.4)	3.1 (5.3)	<0.01
Female sex among FFS Medicare patients, %^f^^,^^g^	54.3 (5.8)	54.3 (5.0)	0.00	54.3 (4.9)	54.3 (4.9)	<0.01
Age among FFS Medicare patients, y^f^^,^^g^	73.1 (4.5)	72.8 (3.5)	0.07	73.2 (4.7)	73.2 (3.4)	<0.01
Charlson Comorbidity Index score among FFS Medicare patients^f^^,^^g^	1.95 (0.34)	2.04 (0.28)	0.29^b^	2.00 (0.31)	2.00 (0.28)	<0.01
Inpatient mortality among FFS Medicare patients^f^^,^^g^	76.4 (82.7)	96.7 (96.9)	0.23^b^	83.0 (86.3)	83.0 (83.0)	<0.01

Abbreviations: FFS, fee-for-service; FTE, full-time equivalents; ICU, intensive care unit; SMD, standardized mean difference.

^a^ Sources include the FFS Medicare inpatient files, hospital cost reports, hospital-submitted case counts Freedom of Information Act request, and Department of Health and Human Services disbursement records.

^b^ *P* < .01.

^c^ *P* < .001.

^d^ *P* < .05.

^e^ Includes only health care provider relief funding administered by the US Department of Health and Human Services. Some other sources of funding (eg, Paycheck Protection Program, Federal Emergency Management Agency grants, American Rescue Plan Act awards) not included.

^f^ 2018 Value included in overlap weighting.

^g^ 2019 Value included in overlap weighting.

^h^ Value and logarithm of value included in overlap weighting.

^i^ Race, ethnicity, and sex data reflect hospital-level information recorded in Medicare inpatient file claims.

Prior research finds that health care provider relief stabilized hospital finances and improved margins for many hospitals.^13,14,15,16,17^ However, a spike in administrative spending^18^ and uneven relationship between correlates of need and relief received^13,19,20,21,22,23^ raise questions about the targeting of relief. In addition, work to date has not documented the association between relief and specific clinical services. Excepting one methodologically oriented working paper focused on only the (smaller) $13 billion SNH pool,^24^ literature has yet to study the marginal dollar of relief.

The impact of this marginal dollar is policy relevant, but not obvious. On the one hand, hospitals receiving extra support from federal programs may have been better able to minimize financial deterioration, add capacity, secure scarce equipment, and recruit and retain needed personnel. On the other hand, resources were sometimes unavailable for purchase at any price. Increased revenues may ultimately have strengthened finances beyond simply holding health care providers financially harmless. Exploiting the strict cutoffs of the SNH and HI funding streams, this article analyzes the extent to which enhanced funding furthered the statute’s aims of both covering increased costs and expanding clinical capacity.

## Methods

### Data

The hospital cost reports used by HHS to determine safety-net funding were obtained from the RAND Hospital Data tool.^25^ Hospital-reported case counts used to determine eligibility for HI funding were obtained from the Health Resources and Services Administration through a Freedom of Information Act request. The cost reports and Freedom of Information Act data were used to replicate HHS’s SNH and HI funding decisions, respectively. This work follows the Strengthening the Reporting of Observational Studies in Epidemiology (STROBE) reporting guideline for cohort studies and was approved by the Harvard Longwood Institutional Review Board.

Outcome data were obtained from fee-for-service (FFS) Medicare inpatient files for 2018 through 2021 and hospital cost reports standardized to each of 2018, 2019, 2020, and 2021.^25^ Records of relief by facility was obtained from public HHS records and characterized following previous work.^19^ The *New York Times* COVID-19 GitHub Repository was used to ascertain cases of COVID-19 by county.^26^ Hospital characteristics and system affiliation were ascertained from hospital cost reports and the 2020 version of the Agency for Healthcare Research and Quality Compendium of US Health Systems.^27^

### Sample

Hospitals with cost report data available for each of 2018 through 2021 were included in the preliminary study population. Hospitals receiving extra rural relief payments were excluded since there were no similar hospitals not receiving these payments. A flowchart and full list of exclusion criteria are provided in eAppendix 3 in Supplement 1.

SNH and HI funding were distributed in multiple waves; recipients of the last wave of HI funding were announced on July 17, 2020. I therefore excluded data from January 1, 2020, and July 30, 2020, as a washout period for the analyses of Medicare inpatient data.

### Independent Variables

The basic regression discontinuity design accommodates 1 running variable that determines receipt of a treatment. However, the treatment considered here—receipt of enhanced relief—was determined by 6 microlevel variables (3 for SNH funding and 3 for HI funding), 2 meso-level variables (1 for SNH funding and 1 for HI funding), and 1 macro-level variable (receipt of SNH or HI funding) (Table 1; eAppendix 1 in Supplement 1).

**Figure 1.**
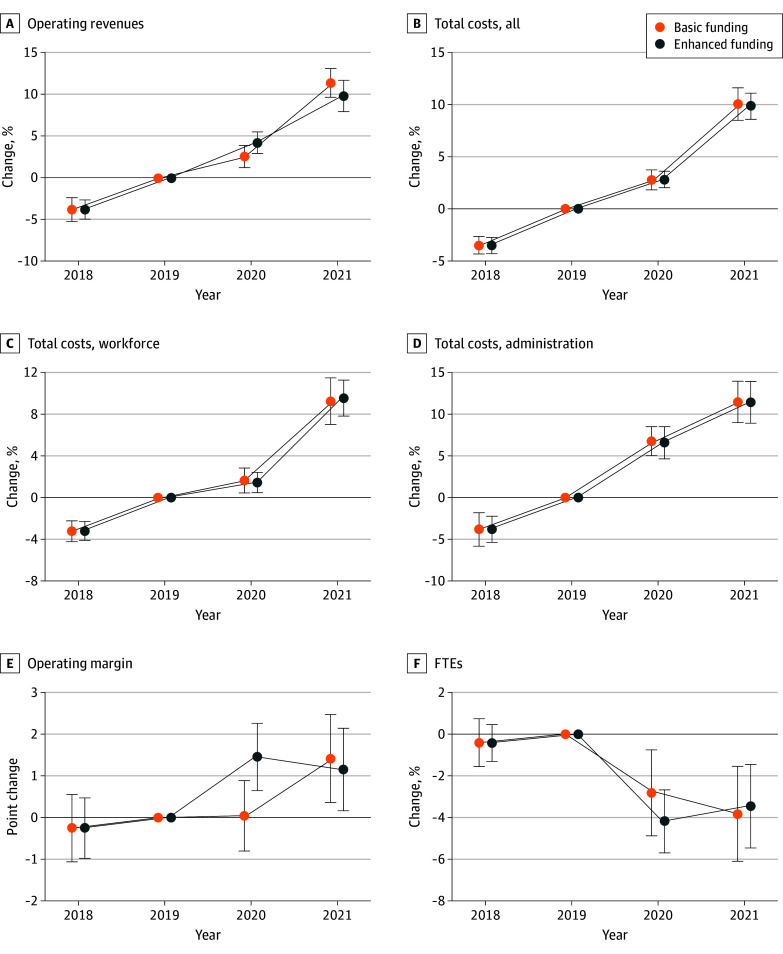
Change in Hospital Revenues, Costs, and Margin Relative to 2019 by Receipt of Enhanced COVID-19 Relief Each panel shows point estimates and 95% CIs for change in outcome across receipt of enhanced relief. Coefficients and estimates should be interpreted relative to 2019 (base year). Point estimates and 95% CIs were derived from weighted event study models with hospital fixed effects. The difference in 2020 operating margin across receipt of enhanced relief was significantly different than 0 notwithstanding the overlap of the 95% CI for enhanced relief and the 95% CI for basic relief. Outcomes are derived from hospital cost reports for 2018 through 2021. Error bars indicate 95% CIs. FTE indicates full-time equivalent.

eAppendix 4 in Supplement 1 details how this setup was accommodated. In brief, I began by normalizing each of the 6 hospital-specific microlevel variables. This enabled the calculation of apples-to-apples distance-to-qualification values. Distances calculated reflect recent recommendations for multiscore regression discontinuity designs,^28^ and accounted for the any of the above and all of the above qualification criteria of the HI and SNH funding streams, respectively (Figure 1; eAppendix 1 in Supplement 1). Positive values implied qualification for HI, SNH, or HI and SNH funding. Negative values implied qualification for neither HI nor SNH funding. All values were expressed in SDs from qualification. The primary analysis was limited to hospitals within 0.5 SD from funding. Sensitivity analyses considered 2 alternate windows.

### Dependent Variables

I analyzed 5 measures related to hospital income: total operating revenues (inclusive of relief funds), total costs, and operating margin. I separately considered costs specific to labor (inclusive of contract labor) and administrative costs as well as full-time equivalents. I also considered 4 balance sheet measures: current assets, current liabilities, cash on hand per bed, and net working capital ratio (ie, liquidity).^29^ Each measure was standardized to the calendar year and windsorized at the 5th and 95th percentiles.

I considered 8 outcomes related to inpatient utilization of FFS Medicare beneficiaries: admission for any cause, admission for COVID-19 (*International Statistical Classification of Diseases, Tenth Revision, Clinical Modification *[*ICD*-*10*-*CM*] code U07.1), hemodialysis during inpatient stay (*International Statistical Classification of Diseases, Tenth Revision, Procedure Coding System *[*ICD*-*10*-*PCS*] codes 5A1D*), mechanical ventilation during inpatient stay (*ICD*-*10*-*PCS* codes 5A09* or 5A19*), admission for lower joint replacement (*ICD*-*10*-*PCS* codes 0SR*), Charlson Comorbidity Index score (calculated using *ICD*-*10*-*CM* admission diagnoses), and death (discharge status code 20).

### Conceptual Strategy

A simple regression of outcomes on receipt of enhanced relief could be confounded by factors influencing both receipt of enhanced relief as well as outcomes. For instance, a high volume of early COVID-19 pandemic cases might tend to increase costs as well as the probability of receiving HI funding.

I used 3 complementary strategies to reduce confounding. First, I calculated hospital-level overlap weights across receipt of enhanced funding. Weighting balanced key observable characteristics across receipt of funding—reducing bias if observable factors were correlated with both receipt of funding and an outcome of interest. Overlap weighting implies that estimates from the outcome model are most credible for hospitals at the edge of treatment—that is, hospitals just as likely to receive enhanced funding as to not receive enhanced funding (eAppendix 5 in Supplement 1).^30^ Second, use of a preperiod—as in a difference-in-differences design—reduced confounding from unobservable but time-invariant hospital-specific factors. Third, reliance on strict administrative thresholds to assign treatment introduced an element of random assignment that might be leveraged in a local randomization analysis of a regression discontinuity design.^28^ This difference-in-discontinuities design has been used elsewhere.^31,32^

### Statistical Analysis

I began by characterizing observable characteristics across receipt of enhanced relief and verified the smoothness of each microlevel running variable across its cutoff (eAppendix 5 in Supplement 1). I next estimated hospital-level overlap weights by predicting the probability of hospital-level enhanced funding using preperiod hospital characteristics plausibly related to financial performance and service delivery (eAppendix 6 in Supplement 1). The characteristics used in weighting are denoted in Table 2. Annual hospital-level expenses, revenues, assets, and liabilities for each of the 2 preperiod years were entered as both levels and logarithms; this implies that units were matched on the magnitude of preperiod growth as well as preperiod levels.^33^ The other variables—which tend to evolve slowly, if at all—were weighted only on level. Excepting region, fiscal year, and for-profit control, the weighting scheme used both 2018 and 2019 values.

The hospital-year combination was the unit of analysis. I first estimated event study models regressing each outcome for hospital *i* in year *t* on the interaction of an indicator for year *t* and an indicator for (eventual) receipt of enhanced relief (eAppendix 7 in Supplement 1). I included hospital and year fixed effects. Data from 2019 were omitted, implying that each term is interpreted relative to 2019. The coefficient corresponding to 2018 provides evidence of the comparability of preperiod trends. I next estimated models that pool the preperiod by omitting the indicator for 2018 as well as the indicator for 2019. The coefficient for 2020 (2021) quantifies expected within-hospital change in the outcome between the baseline period of 2018/2019 and 2020 (2021). I emphasize these coefficients—and the difference between coefficients (termed *contrast*)^34,35^—in the Results.

I also conducted 2 exploratory analyses. First, I investigated the role of for-profit ownership in moderating correlates of relief. The quantity of interest for this analysis was the difference between (1) the association of enhanced relief with the outcome when received by a nonprofit or public hospital and (2) the association of enhanced relief with the outcome when received by a for-profit hospital (eAppendix 8 in Supplement 1). In the second exploratory analysis, I assessed whether membership in a large health system (10 or more hospitals) was associated with detectible differences in outcomes. The quantity of interest was the difference between (1) the association of enhanced relief with the outcome when received by a hospital belonging to a large system and (2) the association of enhanced relief with the outcome when received by an independent or small-system hospital (eAppendix 8 in Supplement 1).

For sensitivity analyses, I (1) repeated the original analysis but adjusted the sample window from 0.5 SD to 0.33 SD, (2) adjusted the sample window from 0.5 SD to 0.67 SD, and (3) omitted overlap weights from estimation. Excepting this third sensitivity analysis, each fixed-effects regression model accounted for hospital-level overlap weights.

I clustered SEs by hospital and present 2-sided 95% CIs to emphasize the range of plausible associations. The absence of zero in the CI was interpreted as statistical significance. There was no adjustment for multiple comparisons. *P* values were calculated using Wald tests for binary variables and χ^2^ tests for categorical variables.

All analyses were completed in R version 4.3 (The R Foundation) with the WeightIt,^36^ fixest,^37^ marginaleffects,^34^ and Charlson^38^ packages.

## Results

### Descriptive Statistics

There were 4795 hospitals with cost report data for each of 2018, 2019, 2020, and 2021. Of these hospitals, 555 geographically dispersed hospitals were included in the primary analysis (Table 2; eAppendix 9 in Supplement 1). Of the 555 hospitals analyzed, 244 received basic relief and 311 received HI funding, SNH funding, or both (Table 2). Before weighting, basic relief hospitals received a mean aid of $7.0 million (about $45 000 per bed) (Table 2; eAppendix 2 in Supplement 1). Enhanced relief hospitals received a mean of about $15.4 million in total aid (about $100 000 per bed) (Table 2; eAppendix 2 in Supplement 1).

Hospitals receiving enhanced funding tended to have higher uncompensated care costs, lower profit margins, and higher disproportionate patient percentages (Table 2). These differences follow mechanically from the SNH funding criteria.

Before weighting, enhanced relief hospitals were also modestly larger in size, personnel, and activity. The share of Black FFS Medicare inpatients served varied more substantially across receipt of enhanced funding before weighting (Table 2). Trends in local disease burden were similar across receipt/nonreceipt of enhanced funding between August 2020 and December 2020 (eAppendix 10 in Supplement 1), but COVID-19 incidence was higher in the counties of enhanced funding during some periods of 2021. Weighting reduced or eliminated what were generally modest differences in preperiod levels and trends.

### Enhanced Relief and Income and Staffing

Operating revenues in 2020 increased over 2018/2019 revenues by 4.5% (95% CI, 3.0-5.9) and 6.1% (95% CI, 4.6-7.6) among basic relief and enhanced relief hospitals, respectively (Figure 1; eAppendix 11 in Supplement 1). This imprecise difference (1.6 points; 95% CI, −0.4 to 3.7) changed direction in 2021 (−1.6 points; 95% CI, −4.3 to 1.2) (Figure 1; eAppendix 11 in Supplement 1).

Costs increased comparably across groups. Moving from the baseline period to 2020 was associated with a 4.5% (95% CI, 3.4-5.7) and 4.6% (95% CI, 3.6-5.6) increase in overall costs for the basic and enhanced relief groups, respectively (Figure 1; eAppendix 11 in Supplement 1). This difference was not significant (contrast of 0.0 points; 95% CI, −1.5 to 1.6) (Figure 1; eAppendix 11 in Supplement 1). Similarly, workforce costs tended to increase by 3.2% (95% CI, 1.8-4.7) and 3.1% (95% CI, 1.8-4.3) in the basic and enhanced groups, respectively (Figure 1; eAppendix 11 in Supplement 1). This difference was not distinguishable from 0 (contrast, 0; 95% CI, −0.2 to 1.7) (Figure 1; eAppendix 11 in Supplement 1). Consistent with this finding, change in full-time equivalents was also statistically indistinguishable across receipt of enhanced relief (increase in association with enhanced relief, −1.4 points; 95% CI, −4.2 to 1.4) (Figure 1; eAppendix 11 in Supplement 1). The subset of costs allocated to administration spiked in 2020 for basic relief hospitals (increase of 8.6%; 95% CI, 6.6-10.6) and enhanced relief hospitals (increase of 8.5%; 95% CI, 6.5-10.5) alike, but—as with labor costs—did not grow detectibly differently across receipt of enhanced relief (contrast of −0.1 points; 95% CI, −2.9 to 2.7) (Figure 1; eAppendix 11 in Supplement 1).

Differential growth in revenues without differential growth in costs manifested in significant differential growth in operating margin. There was a slight increase in margin between the baseline period and 2020 (increase of 0.2 points; 95% CI, −0.6 to 1.0) (Figure 1; eAppendix 11 in Supplement 1) among basic relief hospitals. But margins in the enhanced relief group increased 1.6 points (95% CI, 0.8-2.4) (Figure 1; eAppendix 11 in Supplement 1). This difference of 1.4 points (95% CI, 0.3-2.5) was significantly different than 0 (Figure 1; eAppendix 11 in Supplement 1). The sign of the difference flipped in 2021 (differential decrease in margin of −0.3 points; 95% CI, −1.6 to 1.1) (Figure 1; eAppendix 11 in Supplement 1).

### Enhanced Relief and Liquidity

**Figure 2.**
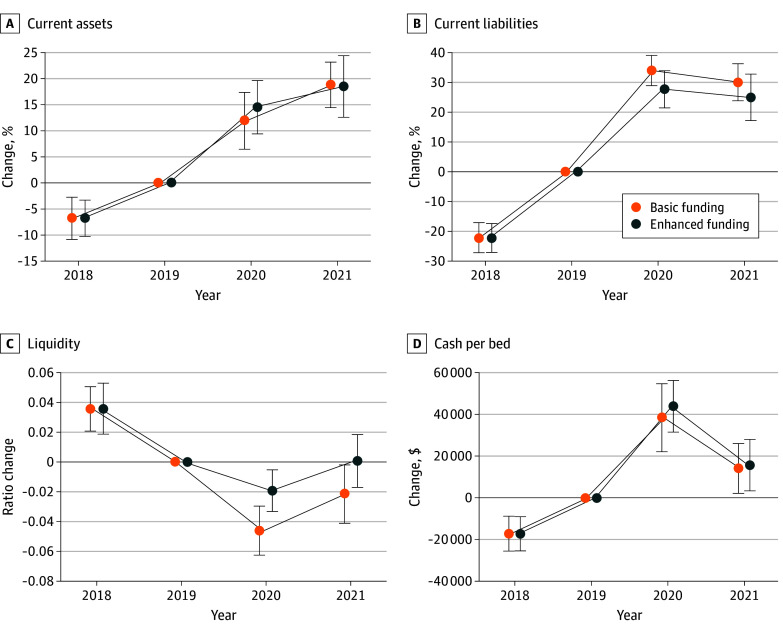
Change in Hospital Assets, Liabilities, Cash, and Liquidity Relative to 2019 by Receipt of Enhanced COVID-19 Relief Each panel shows point estimates and 95% CIs for change in outcome across receipt of enhanced relief. Coefficients and estimates should be interpreted relative to 2019 (base year). Point estimates and 95% CIs were derived from weighted event study models with hospital fixed effects. The difference in 2020 net asset ratio (termed *liquidity*) across receipt of enhanced relief was significantly different than 0 notwithstanding the overlap of the 95% CI for enhanced relief and the 95% CI for basic relief. Outcomes are derived from hospital cost reports for 2018 through 2021. Error bars indicate 95% CIs.

Current assets—and especially current liabilities—increased between the baseline period and 2020 (Figure 2; eAppendix 11 in Supplement 1). However, current assets tended to increase slower than current liabilities. There were smaller increases in total assets among the basic relief hospitals (increase of 15.3%; 95% CI, 9.9-20.8) compared with the enhanced relief hospitals (increase of 17.9%; 95% CI, 12.8-23.0), but the difference was not significant (differential increase of 2.5 points; 95% CI, −4.9 to 10.0) (Figure 2; eAppendix 11 in Supplement 1). The point estimates were reversed for liabilities: total liabilities increased by 45.1% (95% CI, 39.4-50.8) among basic relief hospitals but 38.8% (95% CI, 32.8-44.8) among enhanced relief hospitals for an insignificant difference of −6.3 points (95% CI, −14.6 to 2.0) (Figure 2; eAppendix 11 in Supplement 1). Enhanced funding was not associated with a detectible increase in cash on hand (differential increase of $5460 per bed; 95% CI, −16 406 to 27 327). However, the ratio of assets to liabilities tended to deteriorate less for the enhanced relief group than the basic relief group by about 0.1 SD (differential increase of 0.03 points; 95% CI, 0-0.05) (Figure 2; eAppendix 11 in Supplement 1).

### Enhanced Relief and Medicare Inpatient Volume

**Figure 3.**
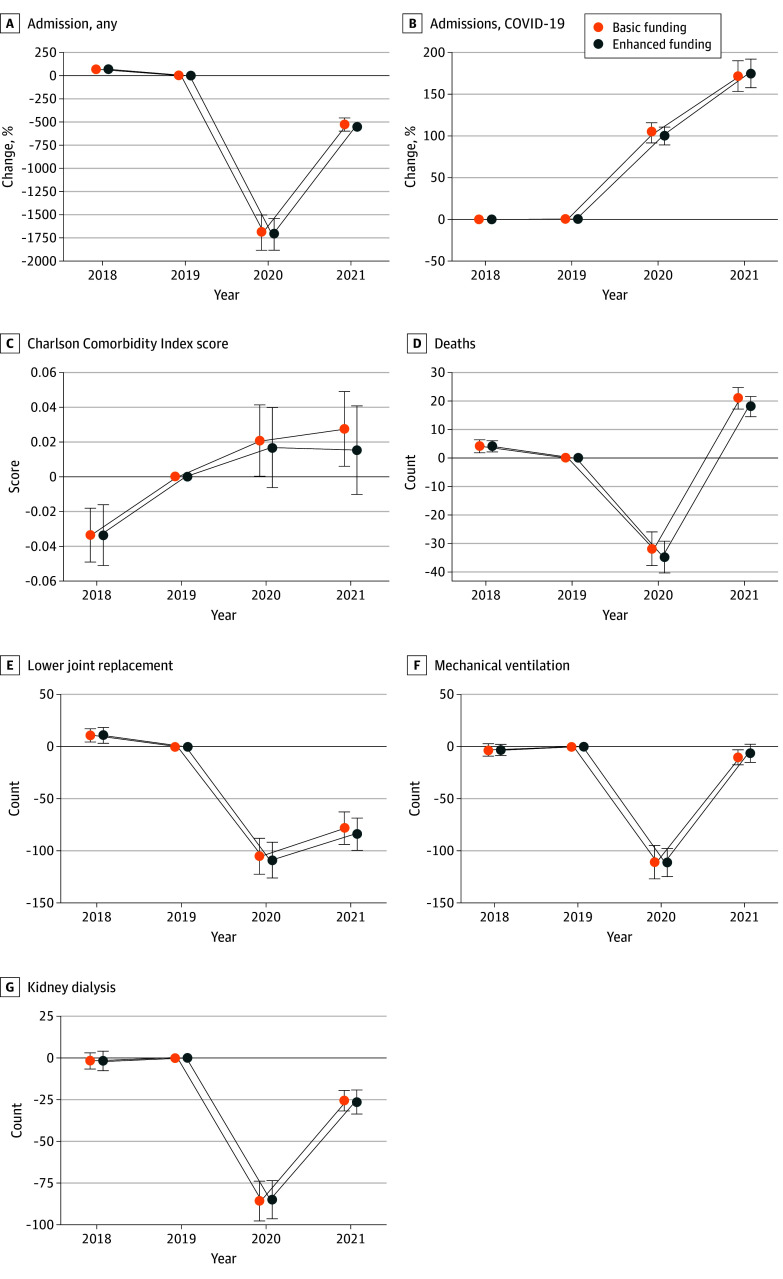
Change in Inpatient Care for Fee-for-Service Medicare Beneficiaries Relative to 2019 by Receipt of Enhanced COVID-19 Relief Each panel shows point estimates and 95% CIs for change in outcome across receipt of enhanced relief. Coefficients and estimates should be interpreted relative to 2019 (base year). Point estimates and 95% CIs derived from weighted event study models with hospital fixed effects. Outcomes are derived from fee-for-service Medicare inpatient files for 2018 through 2021. Error bars indicate 95% CIs.

Inpatient admissions for FFS Medicare beneficiaries changed similarly across receipt of enhanced funding between the preperiod and the final 5 months of 2020 (contrast across receipt of enhanced funding, −19.6 admissions; 95% CI, −281.0 to 241.8) (Figure 3; eAppendix 11 in Supplement 1). In enhanced relief hospitals, the same pattern emerged with respect to FFS Medicare admissions for COVID-19 (−4.0 admissions; 95% CI, −20.0 to 12.0) and lower joint replacement (−3.9 admissions; 95% CI, −29.6 to 21.7) (Figure 3; eAppendix 11 in Supplement 1). Enhanced receipt did not demonstrably affect year-over-year change in inpatient hemodialysis (increase of 0.9 admissions with dialysis; 95% CI, −15.4 to 17.1) or mechanical ventilation (−0.3 services; 95% CI, −20.8 to 20.2) for Medicare FFS beneficiaries (Figure 3; eAppendix 11 in Supplement 1).

The complexity of hospitalized FFS Medicare beneficiaries, as measured by mean Charlson Comorbidity Index score, increased between the preperiod and 2020 in both groups. However, there was no discernable difference in complexity growth across receipt of enhanced funding (differential increase of 0; 95% CI, −0.04 to 0.03) (Figure 3; eAppendix 11 in Supplement 1). There was similarly no identifiable difference in change in inpatient mortality for FFS Medicare inpatients associated with receipt of enhanced relief (−2.9 deaths; 95% CI, −11.3 to 5.5) (Figure 3; eAppendix 11 in Supplement 1).

### Enhanced Relief, Hospital Control, and System Membership

Receipt of enhanced relief was associated with greater workforce expenditures for nonprofit/public hospitals compared with for-profit hospitals (differential increase in expenditures of 1.5 points; 95% CI, 0.1-2.9) (eAppendix 11 in Supplement 1). While 95% CIs included zero, membership in a large system nonsignificantly decreased the estimated association between enhanced relief and workforce expenditures (differential reduction in workforce expenditures of 3.9 points; 95% CI, 0.2-8.1) and current liabilities (differential reduction in total liabilities of 14.4 points; 95% CI, −2.9 to 31.8; eAppendix 11 in Supplement 1). There was little indication of other heterogenous treatment effects across control and system membership (eAppendix 11 in Supplement 1).

### Sensitivity Analyses

Sensitivity analyses that changed the bandwidth from 0.5 SD to 0.33 SD or 0.67 SD but kept overlap weighting produced substantively similar conclusions (eAppendix 11 in Supplement 1). However, results for several claims-derived outcomes were sensitive to the inclusion of overlap weights. In unweighted analyses, enhanced relief was associated with significant differential decreases in overall FFS Medicare admissions notwithstanding significant differential increases in FFS Medicare admissions for COVID-19. Unweighted analyses would also suggest that enhanced relief was associated with differential reductions in inpatient mortality, use of mechanical ventilation, and use of inpatient dialysis among FFS Medicare beneficiaries (eAppendix 11 in Supplement 1).

## Discussion

The US directed billions in COVID-19 relief funding to hospitals beginning in spring 2020. On average, hospitals eligible for HI funding, SNH funding, or both received about twice the relief per bed as otherwise similar hospitals receiving more broadly targeted relief funding. While enhanced funding was associated with insignificantly increased operating revenues of about 2%, extra funding did not lead to detectible differences in spending. There was also little indication that extra funding supported hospitals in pivoting away from elective procedures to provide more resource intensive—but generally less lucrative—medical services. However, enhanced funding was associated with significantly higher operating margins and improved liquidity. While enhanced relief contributed to one part of the Congressional charge for health care provider relief—“reimburs[ing] for lost revenues”^39^—I failed to find evidence that enhanced relief supported health care providers in “prepar[ing] for, and respond[ing] to coronavirus”^39^ beyond the effects of basic relief.

The COVID-19 pandemic–era failure of hospital spending to follow revenues—and resulting improvements in margin—has been documented elsewhere,^13,15,16,17,18^ particularly among rural hospitals,^15,16,18^ public hospitals,^13,15,16,18^ smaller hospitals,^16^ and the most financially vulnerable hospitals.^13,14,15^ At the same time, more general research from the Medicare Payment Advisory Commission^40^ and others^41,42,43^ suggests that hospital expenses and cost structures tend to follow revenues and wealth. However, COVID-19 provider relief was qualitatively different than most other shocks insofar as funding was a one-time resource infusion.

Public and private leaders should consider specific options for reinforcing an acute care system in crisis before the next emergency arrives. A detailed set of plans for supporting an overwhelmed acute care system could complement the recently developed Testing Playbook for Biological Emergencies.^44^ In particular, researchers might model alternative or complementary options for relief distribution, such as dispersing a greater concentration of grant dollars to a smaller number of geographically dispersed quaternary care hospitals or relying on loans convertible to grants in case of severe financial deterioration. Surge capacity could be supported through per diem payments for unoccupied hospital beds; this approach was used in Germany.^45^ Additional conditions could be tied to relief, such as commitment to sharing limited supplies or participation in regional load-balancing efforts. Federal officials might also use funding to support the more rapid deployment of military personnel to civilian settings, to treat civilians in military hospitals, or to facilitate hospital-at-home models.

Better data would likely help irrespective of the particular approach selected. There are severe deficiencies in the quality of hospital-submitted cost reports used to determine both general and targeted distributions.^46,47^ In an audit, the HHS Office of the Inspector General found that obviously erroneous cost report data led to $75 million in hospital-directed overpayments.^48^ A requirement for all hospitals to adopt generally accepted accounting principles might have improved both the accuracy of the general distributions as well as the targeting of SNH funds. Relatedly, HHS used an ad hoc system to collect counts of COVID-19 cases for the HI distributions. Enhanced requirements for the meaningful use of electronic health records could facilitate more expedient allocation of extra support to the most burdened hospitals.

### Limitations

This research has important limitations. First, analyses considered only hospitals near the funding cutoffs. However, even conservative interpretations are still relevant: the 555 hospitals in the main analysis account for more than one-quarter of all US general acute care beds. Second, the analyses of care delivery were limited to FFS Medicare beneficiaries even though beneficiaries with FFS Medicare coverage account for only 20% to 25% of days for the typical hospital. Patterns of care delivery for patients with other coverage might have varied. This limitation may also have limited statistical power to detect changes in care delivery. Third, use of cost report data raises issues related to data quality and reporting practices.^46,47^ I addressed related limitations by (1) weighting on fiscal year, (2) windsorizing extreme values, and (3) accounting for time-invariant idiosyncrasies in cost-reporting practices through hospital fixed effects. Fourth, I accounted for neither variable doses of relief nor relief from other sources (eg, the Paycheck Protection Program, the American Rescue Plan Act, Federal Emergency Management Agency grants, and the COVID-19 Medicare Accelerated and Advance Payments Program) nor possible differences in treatment effects across the 3 treatment conditions (HI, SNH, or HI and SNH funding).^49^ Future work might consider the variable nature of enhanced relief and provide a more holistic accounting of relief funding from various federal funding streams. Fifth, a small share of relief-eligible hospitals refused relief. The intent-to-treat approach used here would tend to bias estimates toward the null. Sixth, this work did not evaluate the impact of relief across the racial, ethnic, and social characteristics of hospital populations. Given documented disparities in outcomes for hospitalized patients^39^—and the possibility for funding to reduce the magnitude of inequities—this is a crucial area for future research.

## Conclusions

The US hospital system will almost certainly face another crisis.^50^ Policymakers should consider optimal approaches to the distribution of emergency aid before the next crisis arrives. Experience with the 2020 targeted funding streams can inform planning to support hospital solvency, maximize the impact of funding on clinical care and operations, and avoid differentially improving finances among similarly situated hospitals.

## Notes

Supplement 1. **eAppendix 1.** Relief Distribution Formulas **eAppendix 2.** Relief Funding, by Distance to Threshold **eAppendix 3.** Study Flowchart **eAppendix 4.** Composite Running Variables **eAppendix 5.** Distribution of Running Variables **eAppendix 6.** Weighting **eAppendix 7.** Estimation **eAppendix 8.** Estimated Impact of Enhanced Relief in 2020 Across For-Profit Control and Large System Membership **eAppendix 9.** Geographic Distribution of Study Hospitals **eAppendix 10.** Area Cases of COVID-19 Cases Across Hospital Receipt of Enhanced Funding **eAppendix 11.** Coefficients and Contrasts Across Specifications

Supplement 2. Data Sharing Statement
