# Supplementary material for: Enhanced COVID-19 Provider Relief, Hospital Finances, and Care for Medicare Inpatients
Source: JAMA Health Forum. 2025 Mar 7;6(3):e250046. doi: 10.1001/jamahealthforum.2025.0046 (PMC11889469; doi:10.1001/jamahealthforum.2025.0046)
Supplement: Supplement 1. — eAppendix 1. Relief Distribution Formulas eAppendix 2. Relief Funding, by Distance to Threshold eAppendix 3. Study Flowchart eAppendix 4. Composite Running Variables eAppendix 5. Distribution of Running Variables eAppendix 6. Weighting eAppendix 7. Estimation eAppendix 8. Estimated Impact of Enhanced Relief in 2020 Across For-Profit Control and Large System Membership eAppendix 9. Geographic Distribution of Study Hospitals eAppendix 10. Area Cases of COVID-19 Cases Across Hospital Receipt of Enhanced Funding eAppendix 11. Coefficients and Contrasts Across Specifications [file jamahealthforum-e250046-s001.pdf]

Supplemental Online Content

Buxbaum JD. Enhanced COVID-19 provider relief, hospital finances, and care for Medicare inpatients. *JAMA Health Forum.* 2025;6(3):e250046. doi:10.1001/jamahealthforum.2025.0046

eAppendix 1. Relief Distribution Formulas
eAppendix 2. Relief Funding, by Distance to Threshold
eAppendix 3. Study Flowchart
eAppendix 4. Composite Running Variables
eAppendix 5. Distribution of Running Variables
eAppendix 6. Weighting
eAppendix 7. Estimation
eAppendix 8. Estimated Impact of Enhanced Relief in 2020 Across For-Profit Control and Large System Membership
eAppendix 9. Geographic Distribution of Study Hospitals
eAppendix 10. Area Cases of COVID-19 Cases Across Hospital Receipt of Enhanced Funding
eAppendix 11. Coefficients and Contrasts Across Specifications

This supplementary material has been provided by the authors to give readers additional information about their work.

© 2025 Buxbaum JD. *JAMA Health Forum*.

eAppendix 1. Relief Distribution Formulas

**Stream**	**Formula**	**Source**
**High-Impact Distributions** $21 billion across 960 hospitals[38–40]	$$max\left[\begin{matrix}\text{HI\_IMPACT\_ROUND\_1}_i\ ,\\ max\left[\begin{matrix}\mathbb{1}(\text{CASES}_{i,t} \geq 161),\\ \mathbb{1}\left(\frac{\text{CASES}_{i,t}}{\text{BEDS}_{i,t}} \geq 0.54864\right)\end{matrix}\right] \times \text{CASES}_{i,t} \times 50{,}000 - \text{HI\_IMPACT\_ROUND\_1}_i\end{matrix}\right]$$ for hospital i where period t spans January 1, 2020, *through June 10, 2020*. CASES is the count of Covid-19 inpatient admissions at hospital i across all payers.BEDS is count of beds at hospital i. $\text{HI\_IMPACT\_ROUND\_1}_\text{i}$ is hospital i's funding from the initial round of high-impact distributions. $\text{HI\_IMPACT\_ROUND\_2}_\text{i}$ is given by: $$\mathbb{1}(\text{CASES}_{i,t'} \geq 100) \times \left(\text{CASES}_{i,t'} \times 76{,}975 + 2 \times 10^9 \times \frac{\text{DSH}_i + \text{UCC}_i}{\sum_{i=1}^{n}\left(\mathbb{1}(\text{CASES}_{i,t'} \geq 100) \times (\text{DSH}_i + \text{UCP}_i)\right)}\right)$$ for hospital i where t' represents January 1, 2020, *through April 10, 2020*. DSH and UCP refer to Medicare disproportionate share payments and uncompensated care payments, respectively. DSH and UCP are from hospital i's most recent cost report as of May 2020.	Hospital-reported counts, as obtained by FOIA request, and hospital cost reports
**Safety-Net Distributions** $13 billion across 899 hospitals[12,39,40]	$$\mathbb{1}(\text{DPP}_i \geq .202) \times \mathbb{1}\left(\frac{\text{UCC}_i}{\text{BEDS}_i} \geq 25{,}000\right) \times \mathbb{1}\left(\min\left(\frac{\text{PROFIT}_{i,t^*} + \text{PROFIT}_{i,t^*-1}}{2}\right) \leq .03\right) \times max\left[\begin{matrix}5{,}000{,}000,\\ min\left[\begin{matrix}50{,}000{,}000\\ \frac{\text{DPP}_i \times \text{BEDS}_i}{\sum_{\text{i}=1}^{\text{n}} \text{DPP}_i \times \text{BEDS}_i}\end{matrix}\right]\end{matrix}\right]$$ for hospital i. DPP, UCC, and PROFIT refer to Medicare disproportionate patient percentage, uncompensated care costs, and profit margin, respectively.All are taken from the hospital i's most recent cost report as of May 2020.n is the count of hospitals meeting all three threshold criteria.t* is any of hospital i's most recent four cost reports.	Hospital cost reports

© 2025 Buxbaum JD. *JAMA Health Forum*.

eAppendix 2. Relief Funding, by Distance to Threshold

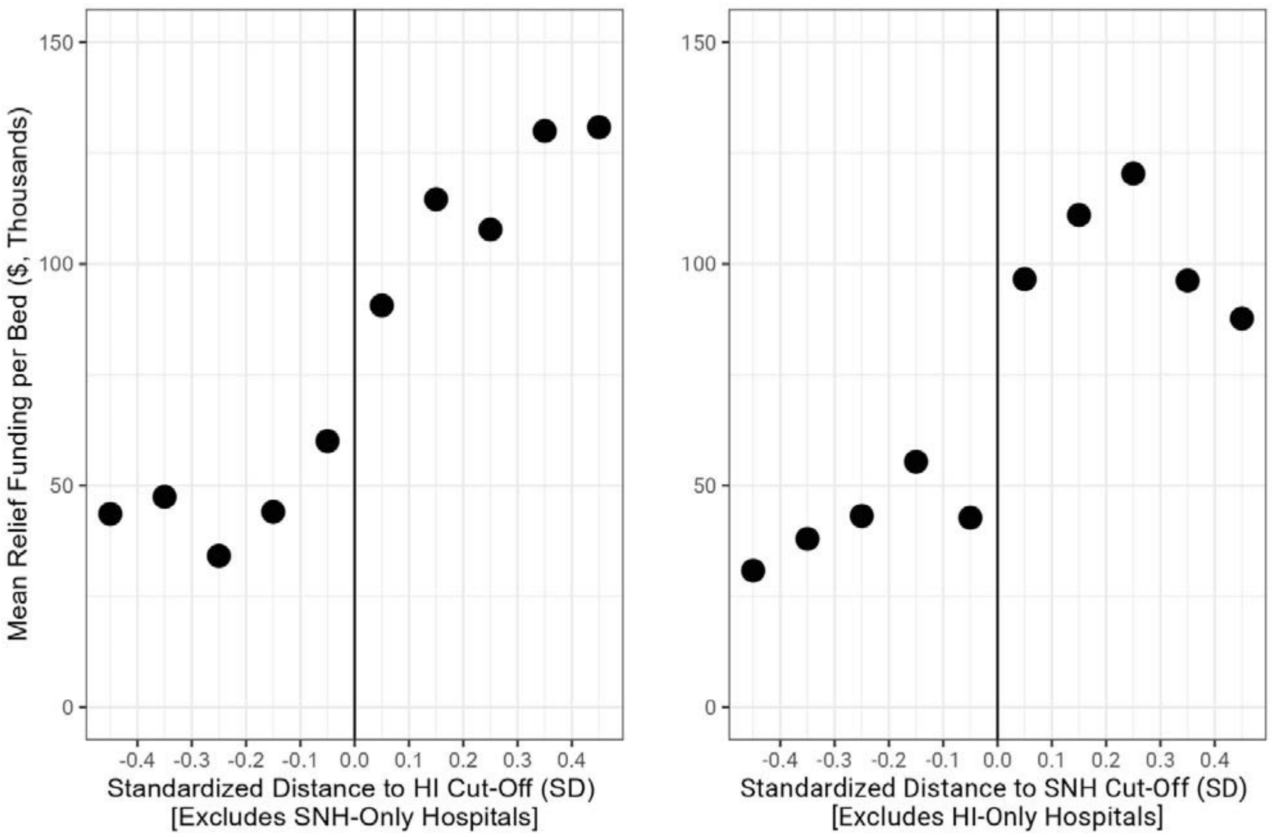

SOURCES: HHS Provider Relief Disbursement Records, hospital cost reports, FOIA request of hospital-reported case counts

© 2025 Buxbaum JD. *JAMA Health Forum*.

eAppendix 3. Study Flowchart

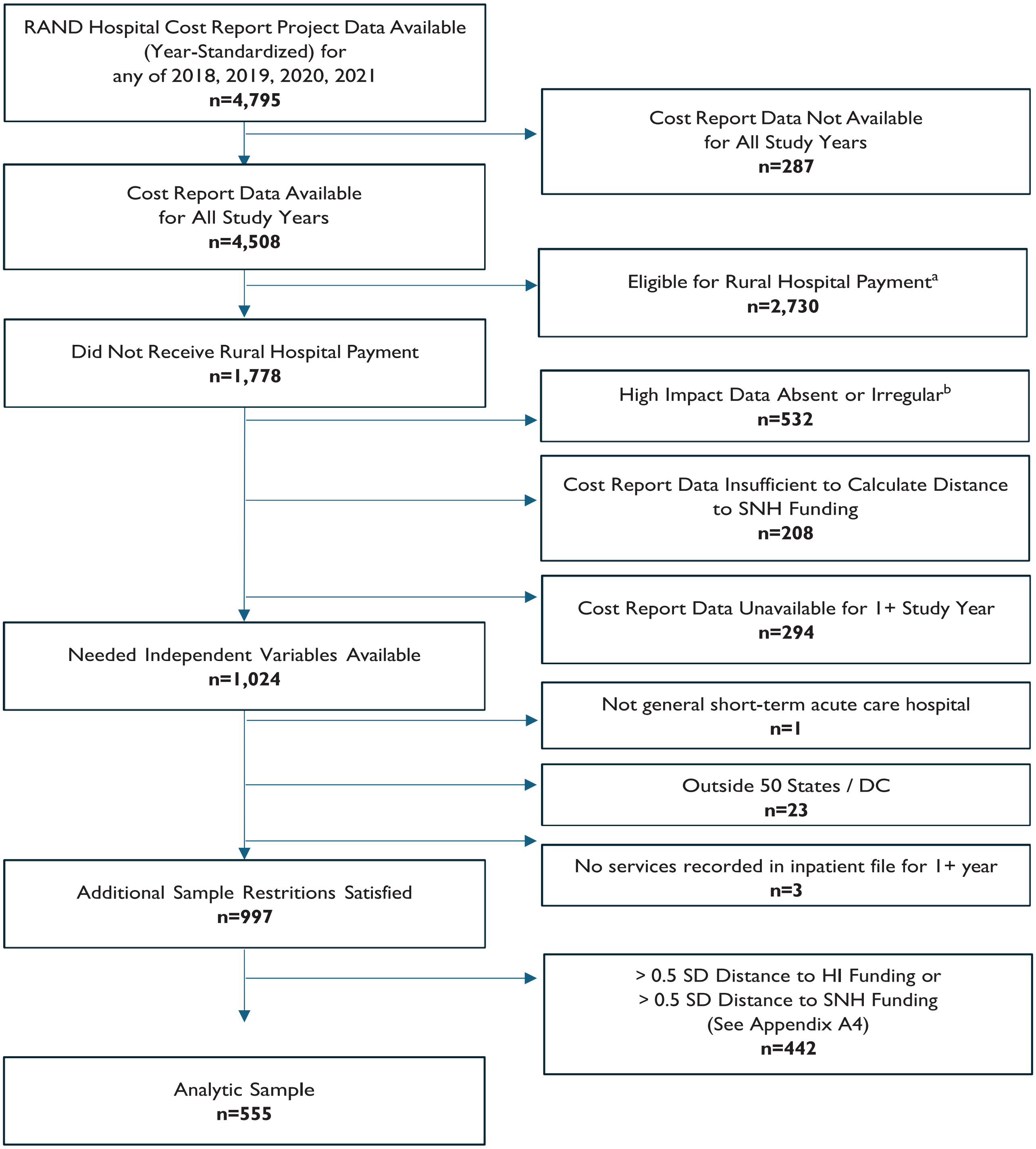

[a] I excluded rural hospitals because there were no similar hospitals that did *not* receive enhanced funding. Hospitals meeting one or more of the following criteria were eligible for rural payments: designation as a critical access hospital, sole community hospital, Medicare dependent hospital, or rural referral center (if located in a small metropolitan area); location in a non-metropolitan county; location in any of 132 low-density census tracts; location in a rural-urban commuting area (RUCA) with a code greater than 3; and/or location 40 or more miles away from another hospital. See HRSA guidance (see reference 12 in main text).
[b] For example, more cases reported through April 10 than reported through June 10

© 2025 Buxbaum JD. *JAMA Health Forum*.

eAppendix 4. Composite Running Variables

**Scenario**	**Rules**
"Any" Criteria<br>(High-Impact Funding)	(1) If all three micro-level running variables are positive, calculate the Euclidean distance between the three values and the origin.<br>(2) If two of three micro-level running variables are positive, take the Euclidean distance between the two positive values and the origin.<br>(3) If one of the three micro-level running variables is positive, select that value.<br>(4) If none of the three micro-level running variables are positive, select the maximum value.
"All" Criteria<br>(Safety-Net Funding)	(1) If all three micro-level running variables are positive, select the minimum of all three values.<br>(2) If two of three micro-level running variables are positive, select the minimum of all three values.<br>(3) If one of the three micro-level running variables is positive, calculate the product of -1 and the Euclidean distance between the origin and the two negative values.<br>(4) If none of the three micro-level running variables are positive, take the product of -1 and the Euclidean distance between the origin and all three values.

This approach was adapted from the recommendations at page 88 of:

Cattaneo MD, Idrobo N, Titiunik R. A practical introduction to regression discontinuity designs: Extensions. arXiv preprint arXiv:2301.08958. 2023 Jan 21. Available at: https://arxiv.org/abs/2301.08958

© 2025 Buxbaum JD. *JAMA Health Forum*.

eAppendix 5. Distribution of Running Variables

**Funding Stream and Running Variable**	**Distribution of Hospitals Across Thresholds**
**Safety-Net Funding**<br>*Meet all criteria*	
Uncompensated care per bed >= $25,000	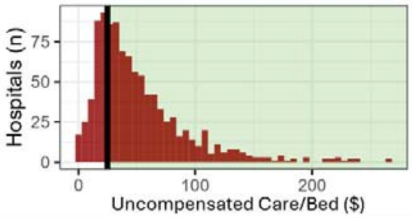
Disproportionate patient percentage >= 20.2%	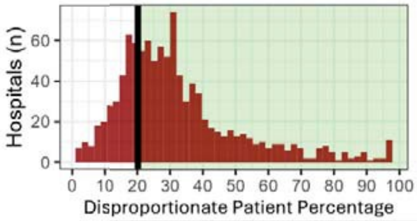
Profit < 3% in most recent year or mean profit <3% over 2 consecutive years in last 5 years	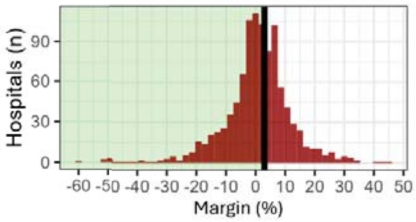
**High-Impact Funding**<br>*Meet any criterion*	
Covid-19 cases through April 10, 2020 >=100	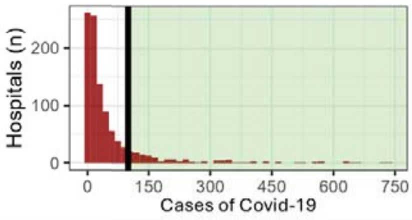
Covid-19 cases per bed through June 10, 2020 >=0.54864	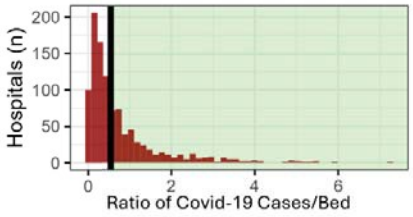
Covid-19 cases through June 10, 2020 >=161	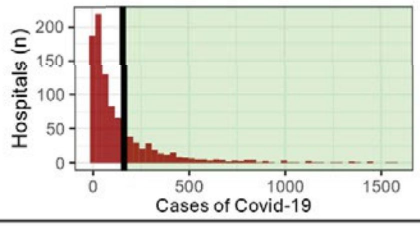

Data from Health Resources and Services Administration and hospital cost reports. Disproportionate patient percentage is the sum of (a) the percentage of inpatient days attributable to patients with Medicaid coverage but *not* Medicare Part A, and (b) the percentage of inpatient days for patients with Medicare Part A receiving supplemental security income (SSI).

© 2025 Buxbaum JD. *JAMA Health Forum*.

eAppendix 6. Weighting

Approach. In overlap weighting, untreated units receive the propensity score as their weight. I used logistic regression to predict the probability of treatment (reciept of enhanced relief) for hospitals within 0.5 standard deviations of the criteria for funding:

$(\text{logit}\ (\text{CONTROL}_i, \text{FY}_i,\ \log\ (\text{OPERATING\_REVS}_{i,\,2016-2019}),\ \log\ (\text{OPERATING\_REVS}_{i,\,2020})\ \ldots)3$

Treated units receive a weight of 1 minus the propensity score. Variables included in the propensity score are listed in Table 2 of the main text. I estimated overlap weights using WeightIt for R.

A model estimated with overlap weights gives the most influence to units "at the edge" of treatment. This leads to treated units with very high probability of treatment receiving relatively little influence, and vice versa. The resulting estimand applies to a hypothetical unit that is equally likely to receive or not receive treatment (units at "clinical equipoise").

Inverse propensity treatment weighting (IPTW) also uses PSs. However, calculation of the weights differs. In IPTW, treated units are assigned a weight of the reciprocal of the PS (i.e., 1/PS). Untreated units are assigned the reciprocal of 1 minus the propensity score (i.e., 1/(1-PS). The target estimand in an outcome model estimated with IPTW weights is typically the average effect of treatment in the entirety of the sample (ATE) or the average effect of treatment among the treated (ATT). I use overlap weights because the estimand – the average effect of treatment for units at the edge of qualification (average treatment for the overlap population, or ATO) – aligns well with the regression discontinuity component of the identification strategy. The figure below shows the distribution of overlap weights.

Appropriateness. Use of pre-period outcomes in matching – which might be conceptualized as weighting with values of only zero or one – has been scrutinized. Daw and Hatfield demonstrate that matching can increase bias in difference-in-difference strategies when units to be matched come from different populations.[a] However, Ryan shows that matching on pre-period outcomes can reduce bias when treatment is correlated with pre-period trends *and* units are drawn from one population.[b] The sharp cut-offs used to determine qualification for enhanced relief increase the plausibility of the assumption that "barely surpassed" and "barely missed" hospitals were drawn from one population – not two populations. Therefore, the Ryan scenario is more relevant than the Daw/Hatfield scenario for the current work. Recognizing that both weighting and matching are forms of pre-processing, weighting on lagged pre-period outcome levels is likely to reduce bias in this study set-up. However, the final sensitivity analysis shows that findings are reasonably robust to the inclusion or exclusion of weights.

[a] Daw JR, Hatfield LA. Matching and Regression to the Mean in Difference-in-Differences Analysis. Health Serv Res. 2018;53(6):4138-56.

[b] Ryan AM. Well-Balanced or too Matchy-Matchy? The Controversy over Matching in Difference-in-Differences. Health Serv Res. 2018 Dec;53(6):4106-10.

© 2025 Buxbaum JD. *JAMA Health Forum*.

eAppendix 7. Estimation

I first estimated event study models of the form:

$$\begin{aligned} Y_{i,t} = \\ \beta_1\, I\,(YEAR_t = 2018) \times \mathbb{1}\,(ENHANCED_i) + \\ \beta_2\, I\,(YEAR_t = 2020) \times \mathbb{1}\,(ENHANCED_i) + \\ \beta_3\, I\,(YEAR_t = 2021) \times \mathbb{1}\,(ENHANCED_i) + \\ HOSPITAL_i + YEAR_t + \epsilon_{\mathrm{i,t}} \end{aligned}$$

where $\mathrm{ENHANCED_i}$ denotes (eventual) receipt of enhanced relief by hospital i. Six outcomes – operating revenues, total costs, workforce costs, administrative costs, current assets, and current liabilities – were entered after log transformation. The year 2019 is omitted, implying that each of β_1 through β_3 is interpreted relative to 2019. β_1 provides information on pre-intervention trends. $\mathrm{HOSPITAL_i}$ and $\mathrm{YEAR_t}$ are fixed effects for hospital and year, respectively. Inclusion of hospital fixed effects implies that estimates reflect expected within-hospital change. I apply hospital-level overlap weights and cluster standard errors by hospital. Results from these models are shown in the event-study plots of Figures 2, 3, and 4.

The main analysis presents results from a series of models omitting β_1. The baseline period for these estimates is 2018/2019.

© 2025 Buxbaum JD. *JAMA Health Forum*.

eAppendix 8. Estimated Impact of Enhanced Relief in 2020 Across For-Profit Control and Large System Membership

Control. I began by re-estimating overlap weights within two stratums: hospitals with private control and hospitals with not-for-profit or public control. In addition to the indicator for for-profit control, I excluded measures of discharge equivalents and the claims-derived measures since the logistic model did not converge with the full set of variables. I then estimated this weighted linear model:

$$Y_{i,t} = \beta_1 \mathbb{1}(YEAR_t = 2020) \times \mathbb{1}(ENHANCED_i) + \beta_2 \mathbb{1}(YEAR_t = 2021) \times \mathbb{1}(ENHANCED_i) + \beta_3 \mathbb{1}(YEAR_t = 2020) \times \mathbb{1}(ENHANCED_i) \times \mathbb{1}(FOR\_PROFIT_i) + \beta_4 \mathbb{1}(YEAR_t = 2021) \times \mathbb{1}(ENHANCED_i) \times \mathbb{1}(FOR\_PROFIT_i) + \beta_5 \mathbb{1}(YEAR_t = 2020) \times \mathbb{1}(FOR\_PROFIT_i) + \beta_6 \mathbb{1}(YEAR_t = 2021) \times \mathbb{1}(FOR\_PROFIT_i) + HOSPITAL_i + YEAR_t + \epsilon_{i,t}$$

SEs were again clustered by hospital. I then used the marginaleffects package to characterize the marginal effect of for-profit control on the effect of enhanced relief in 2020.

Large system membership. I began by estimating weights across hospital membership in a system of at least 10 hospitals. Models converged when using the full set of variables denoted in Table 1. I then proceeded as above, but substituting $I\ (LARGE\_SYS_i)$ for $I\ (NFP\_PUBLIC_i)$.

Impact of Enhanced Relief Across Control, 2020

	**Contrast across membership in large system**	**Contrast across for-profit control**
Administrative costs	-2.1 (-8.5, 4.3)	0.2 (-2.6, 3)
Admission*	-52.3 (-629.4, 524.8)	21.1 (-201.4, 243.8)
Cash/Bed	-15110 (-61404, 31184)	-5014 (-22264, 12235)
Charlson score*	-0.01 (-0.08, 0.06)	0 (-0.02, 0.03)
Covid-19 admission*	9.6 (-28.2, 47.3)	0.5 (-12.3, 13.2)
Current assets	-3.4 (-20.6, 13.8)	-3.1 (-8.7, 2.5)
Current liabilities	-14.4 (-31.8, 2.9)	-4.1 (-11, 2.7)
Employees (FTEs)	-3.9 (-9.7, 1.9)	-1.4 (-3.5, 0.8)
Hemodialysis during admission*	4 (-33.3, 41.3)	4.8 (-9.1, 18.6)
In-hospital death*	0.3 (-16.9, 17.5)	-2 (-9, 5.1)
Liquidity	0.03 (-0.03, 0.08)	0 (-0.05, 0.06)
Mechanical ventilation during admission*	0.5 (-46.9, 47.8)	-5 (-22.3, 12.4)
Operating margin (%)	0 (-0.1, 0)	-0.1 (-1, 0.8)
Operating revenues	-4.5 (-10.1, 1)	-0.9 (-2.3, 0.6)
Replacement of lower joint admission*	-3.9 (-60.6, 52.9)	2 (-20, 24)
Total costs	-2.8 (-6, 0.5)	-0.6 (-1.8, 0.6)
Workforce expenditures	-3.9 (-8.1, 0.2)	-1.5 (-2.9, -0.1)

© 2025 Buxbaum JD. *JAMA Health Forum*.

eAppendix 9. Geographic Distribution of Study Hospitals

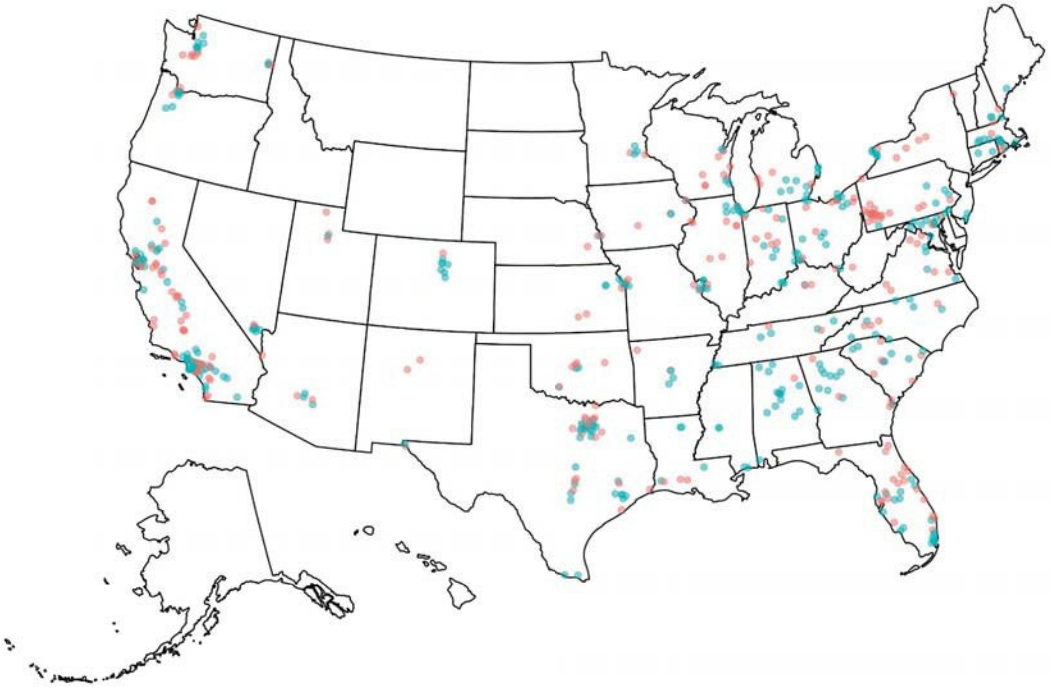

NOTES: Blue dots correspond to in-sample enhanced relief hospitals; red dots correspond to in-sample basic relief hospitals.

© 2025 Buxbaum JD. *JAMA Health Forum*.

eAppendix 10. Area Cases of COVID-19 Cases Across Hospital Receipt of Enhanced Funding

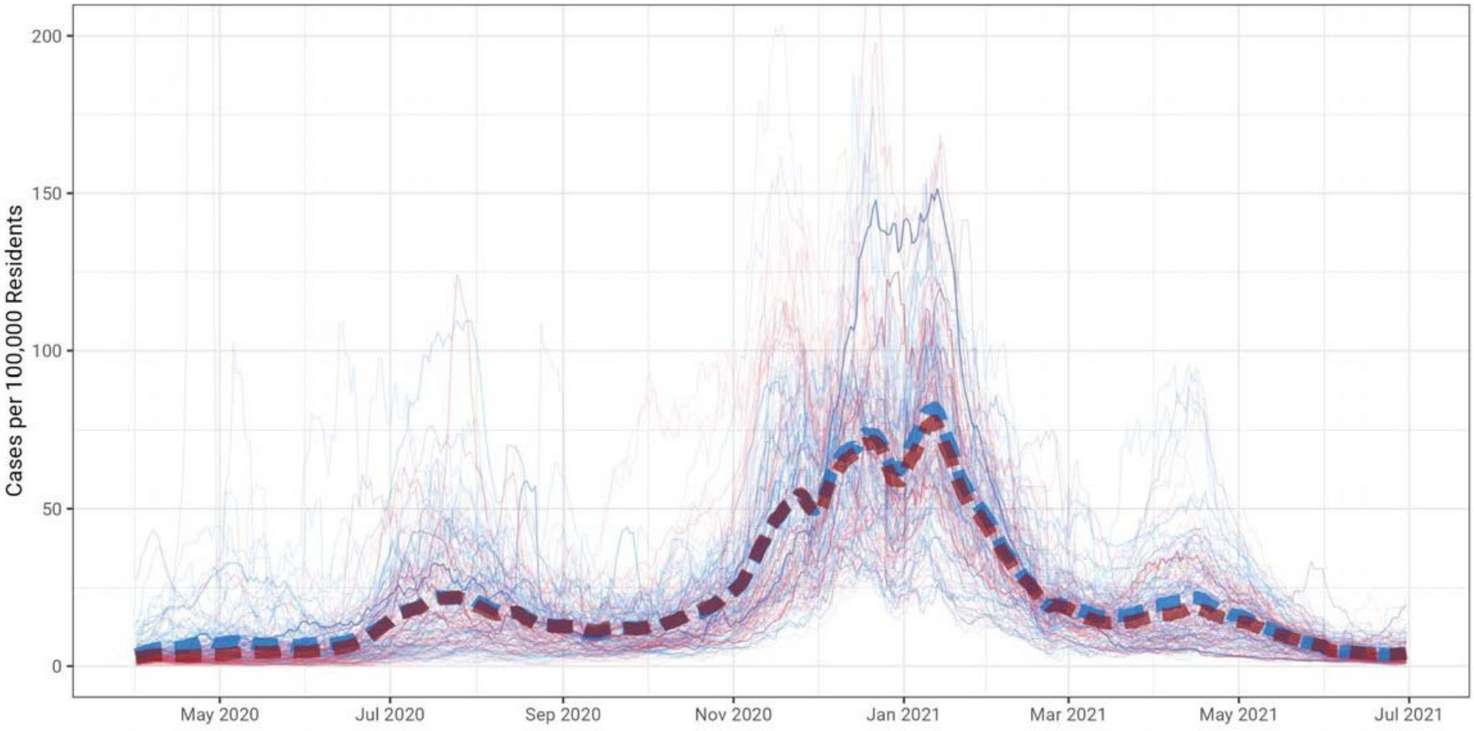

NOTES: Thin lines depict daily count of cases of Covid-19 by county of hospital by receipt/non-receipt of enhanced funding (blue lines and red lines, respectively). Only hospitals within 0.5 SD of receiving safety-net funding and 0.5 SD of receiving high-impact funding ae shown. Dashed lines reflect weighted averages. SOURCE: New York Times Covid-19 GitHub repository.

© 2025 Buxbaum JD. *JAMA Health Forum*.

eAppendix 11. Coefficients and Contrasts Across Specifications

Specification	Year	Basic Relief	Enhanced Relief	Contrast
**Admission (Any)**				
1. One-Half SD, Weighted	2018/2019	Ref	Ref	NA
(Primary Specification)	2020	-1726.2 (-1922, -1530.5)	-1745.8 (-1919, -1572.7)	-19.6 (-281, 241.8)
	2021	-564.6 (-638.1, -491)	-587.5 (-653.3, -521.7)	-22.9 (-121.6, 75.7)
2. One-Third SD, Weighted	2018/2019	Ref	Ref	NA
	2020	-1660.4 (-1946.1, -1374.7)	-1655.7 (-1920.1, -1391.2)	4.7 (-384.6, 394)
	2021	-557.1 (-668.5, -445.8)	-566.6 (-650.3, -482.9)	-9.5 (-148.8, 129.8)
3. Two-Thirds SD, Weighted	2018/2019	Ref	Ref	NA
	2020	-1656.1 (-1829.6, -1482.5)	-1668.1 (-1830.7, -1505.5)	-12 (-249.9, 225.8)
	2021	-537.3 (-602.5, -472.1)	-546 (-603.9, -488.1)	-8.7 (-95.8, 78.5)
4. One-Half SD, Unweighted	2018/2019	Ref	Ref	NA
	2020	-1602.4 (-1764.5, -1440.4)	-1967 (-2147.8, -1786.2)	-364.6 (-607.4, -121.7)
	2021	-514.3 (-573, -455.7)	-668.5 (-740.4, -596.7)	-154.2 (-246.9, -61.5)
**Admission (Covid-19)**				
1. One-Half SD, Weighted	2018/2019	Ref	Ref	NA
(Primary Specification)	2020	103.6 (91.6, 115.6)	99.7 (89.1, 110.3)	-4 (-20, 12)
	2021	171.3 (152.9, 189.6)	174.3 (157.5, 191.1)	3 (-21.9, 27.9)
2. One-Third SD, Weighted	2018/2019	Ref	Ref	NA
	2020	107.9 (87.7, 128.1)	94 (79.3, 108.8)	-13.9 (-38.9, 11.1)
	2021	176.8 (148.3, 205.3)	164.6 (134.8, 194.5)	-12.2 (-53.5, 29.1)
3. Two-Thirds SD, Weighted	2018/2019	Ref	Ref	NA
	2020	99.2 (88.4, 109.9)	95.3 (85.2, 105.3)	-3.9 (-18.6, 10.8)
	2021	163.4 (147, 179.8)	164.3 (148.5, 180.1)	0.9 (-21.9, 23.7)
4. One-Half SD, Unweighted	2018/2019	Ref	Ref	NA
	2020	94.3 (84.2, 104.4)	111.3 (101, 121.6)	16.9 (2.5, 31.4)
	2021	158.8 (143, 174.5)	187.9 (171.6, 204.3)	29.2 (6.5, 51.9)
**Cash per Bed (Dollar Change)**				
1. One-Half SD, Weighted	2018/2019	Ref	Ref	NA
(Primary Specification)	2020	47069.8 (30391.6, 63748.1)	52530 (38388.5, 66671.5)	5460.1 (-16406.4, 27326.7)
	2021	22746.9 (10951.3, 34542.5)	24298 (11493.7, 37102.3)	1551.1 (-15858.2, 18960.5)
2. One-Third SD, Weighted	2018/2019	Ref	Ref	NA
	2020	48642.3 (14916.2, 82368.5)	56852.2 (33135.5, 80568.9)	8209.9 (-33020.4, 49440.1)
	2021	29128.9 (1464.4, 56793.5)	18501 (-6423.4, 43425.5)	-10627.9 (-47864.4, 26608.6)
3. Two-Thirds SD, Weighted	2018/2019	Ref	Ref	NA
	2020	45541.4 (30618.4, 60464.4)	41671.3 (29171.6, 54171.1)	-3870.1 (-23336.5, 15596.4)
	2021	22158.2 (11156.9, 33159.5)	18114.2 (6185.4, 30042.9)	-4044 (-20271.3, 12183.2)
4. One-Half SD, Unweighted	2018/2019	Ref	Ref	NA
	2020	42773.4 (30075.9, 55470.8)	51936.7 (39793.3, 64080)	9163.3 (-8406.2, 26732.8)
	2021	18797.2 (7778.7, 29815.7)	27001 (15639, 38362.9)	8203.8 (-7623.4, 24031)
**Charlson Score**				
1. One-Half SD, Weighted	2018/2019	Ref	Ref	NA
(Primary Specification)	2020	0.04 (0.02, 0.06)	0.03 (0.01, 0.06)	0 (-0.04, 0.03)
	2021	0.04 (0.02, 0.07)	0.03 (0.01, 0.06)	-0.01 (-0.05, 0.02)
2. One-Third SD, Weighted	2018/2019	Ref	Ref	NA
	2020	0.04 (0.01, 0.08)	0.04 (0, 0.07)	-0.01 (-0.06, 0.04)
	2021	0.04 (0.01, 0.08)	0.03 (-0.01, 0.06)	-0.02 (-0.07, 0.04)
3. Two-Thirds SD, Weighted	2018/2019	Ref	Ref	NA
	2020	0.04 (0.02, 0.06)	0.04 (0.02, 0.07)	0 (-0.03, 0.03)
	2021	0.05 (0.02, 0.07)	0.04 (0.02, 0.07)	0 (-0.04, 0.03)
4. One-Half SD, Unweighted	2018/2019	Ref	Ref	NA
	2020	0.04 (0.02, 0.06)	0.02 (0, 0.04)	-0.02 (-0.05, 0.01)
	2021	0.05 (0.03, 0.06)	0.03 (0.01, 0.05)	-0.02 (-0.05, 0.01)

© 2025 Buxbaum JD. *JAMA Health Forum*.

**Current Assets (Percent Change)**				
1. One-Half SD, Weighted	2018/2019	Ref	Ref	NA
(Primary Specification)	2020	15.3 (9.9, 20.8)	17.9 (12.8, 23)	2.5 (-4.9, 10)
	2021	22.2 (18, 26.3)	21.9 (16, 27.7)	-0.3 (-7.5, 6.9)
2. One-Third SD, Weighted	2018/2019	Ref	Ref	NA
	2020	15.1 (5.4, 24.8)	18.9 (12.2, 25.5)	3.7 (-8, 15.5)
	2021	23.4 (15.7, 31.1)	21.8 (13, 30.6)	-1.6 (-13.3, 10.1)
3. Two-Thirds SD, Weighted	2018/2019	Ref	Ref	NA
	2020	13.9 (8.6, 19.1)	15.5 (10.5, 20.6)	1.7 (-5.6, 9)
	2021	21.4 (17.2, 25.6)	18.2 (11.6, 24.7)	-3.2 (-11, 4.6)
4. One-Half SD, Unweighted	2018/2019	Ref	Ref	NA
	2020	14 (9.5, 18.6)	18.3 (14.5, 22.2)	4.3 (-1.7, 10.3)
	2021	20 (15.7, 24.3)	23.6 (19, 28.3)	3.6 (-2.7, 10)
**Current Liabilities (Percent Change)**				
1. One-Half SD, Weighted	2018/2019	Ref	Ref	NA
(Primary Specification)	2020	45.1 (39.4, 50.8)	38.8 (32.8, 44.8)	-6.3 (-14.6, 2)
	2021	41.1 (34.5, 47.8)	36.1 (28.6, 43.6)	-5 (-15, 5)
2. One-Third SD, Weighted	2018/2019	Ref	Ref	NA
	2020	43 (34, 51.9)	37.5 (30.9, 44.1)	-5.4 (-16.6, 5.7)
	2021	38.8 (30.1, 47.6)	38.6 (29.1, 48.1)	-0.2 (-13.2, 12.7)
3. Two-Thirds SD, Weighted	2018/2019	Ref	Ref	NA
	2020	41.5 (35.9, 47.2)	37.6 (32.3, 43)	-3.9 (-11.7, 3.9)
	2021	37.8 (31.7, 43.9)	35.7 (29.2, 42.2)	-2.2 (-11.1, 6.8)
4. One-Half SD, Unweighted	2018/2019	Ref	Ref	NA
	2020	45.9 (41.1, 50.7)	39.3 (34.5, 44)	-6.6 (-13.3, 0.1)
	2021	40.8 (35.2, 46.4)	36.8 (30.8, 42.7)	-4 (-12.2, 4.2)
**Deaths (Count)**				
1. One-Half SD, Weighted	2018/2019	Ref	Ref	NA
(Primary Specification)	2020	-33.9 (-40.1, -27.7)	-36.8 (-42.4, -31.1)	-2.9 (-11.3, 5.5)
	2021	18.8 (15.3, 22.4)	16.1 (12.6, 19.5)	-2.8 (-7.7, 2.2)
2. One-Third SD, Weighted	2018/2019	Ref	Ref	NA
	2020	-29.9 (-38.7, -21.2)	-34.7 (-43.5, -26)	-4.8 (-17.2, 7.6)
	2021	21.5 (16.1, 26.9)	16.6 (11, 22.2)	-4.9 (-12.7, 2.9)
3. Two-Thirds SD, Weighted	2018/2019	Ref	Ref	NA
	2020	-32 (-37.2, -26.7)	-34.4 (-39.4, -29.5)	-2.5 (-9.7, 4.8)
	2021	17 (13.8, 20.2)	14.3 (11.3, 17.4)	-2.7 (-7.1, 1.8)
4. One-Half SD, Unweighted	2018/2019	Ref	Ref	NA
	2020	-32 (-37.4, -26.7)	-42.8 (-48.5, -37.1)	-10.7 (-18.6, -2.9)
	2021	17.1 (13.9, 20.3)	16.9 (13.6, 20.2)	-0.2 (-4.9, 4.4)
**FTEs (Percent Change)**				
1. One-Half SD, Weighted	2018/2019	Ref	Ref	NA
(Primary Specification)	2020	-2.6 (-4.8, -0.4)	-4 (-5.7, -2.3)	-1.4 (-4.2, 1.4)
	2021	-3.6 (-6.1, -1.2)	-3.2 (-5.4, -1.1)	0.4 (-2.9, 3.7)
2. One-Third SD, Weighted	2018/2019	Ref	Ref	NA
	2020	-3.1 (-6.4, 0.2)	-4.9 (-7.7, -2.1)	-1.8 (-6.1, 2.6)
	2021	-5.3 (-9.6, -0.9)	-5.2 (-8.6, -1.8)	0.1 (-5.4, 5.6)
3. Two-Thirds SD, Weighted	2018/2019	Ref	Ref	NA
	2020	-2.2 (-4.3, -0.2)	-3.8 (-5.2, -2.3)	-1.5 (-4, 1)
	2021	-3 (-5.3, -0.7)	-3.3 (-5.2, -1.4)	-0.3 (-3.2, 2.6)
4. One-Half SD, Unweighted	2018/2019	Ref	Ref	NA
	2020	0.2 (-4, 4.4)	-3.5 (-4.8, -2.2)	-3.7 (-8.1, 0.7)
	2021	-3 (-5.3, -0.6)	-3 (-4.7, -1.3)	0 (-3, 2.9)
**Liquidity (Change in Net Asset Ratio)**				
1. One-Half SD, Weighted	2018/2019	Ref	Ref	NA
(Primary Specification)	2020	-0.06 (-0.08, -0.05)	-0.04 (-0.05, -0.02)	0.03 (0, 0.05)

© 2025 Buxbaum JD. *JAMA Health Forum*.

	2021	-0.04 (-0.06, -0.02)	-0.02 (-0.04, 0)	0.02 (-0.01, 0.05)
2. One-Third SD, Weighted	2018/2019	Ref	Ref	NA
	2020	-0.05 (-0.08, -0.03)	-0.03 (-0.05, -0.01)	0.03 (0, 0.06)
	2021	-0.03 (-0.06, 0)	-0.03 (-0.05, -0.01)	0 (-0.04, 0.04)
3. Two-Thirds SD, Weighted	2018/2019	Ref	Ref	NA
	2020	-0.06 (-0.08, -0.04)	-0.04 (-0.05, -0.02)	0.02 (0, 0.04)
	2021	-0.03 (-0.05, -0.01)	-0.02 (-0.04, 0)	0.01 (-0.02, 0.04)
4. One-Half SD, Unweighted	2018/2019	Ref	Ref	NA
	2020	-0.06 (-0.08, -0.05)	-0.04 (-0.05, -0.02)	0.03 (0.01, 0.05)
	2021	-0.04 (-0.06, -0.02)	-0.01 (-0.03, 0)	0.03 (0, 0.05)
**Lower Joint Replacement (Count)**				
1. One-Half SD, Weighted	2018/2019	Ref	Ref	NA
(Primary Specification)	2020	-110.6 (-128.7, -92.6)	-114.6 (-132.9, -96.3)	-3.9 (-29.6, 21.7)
	2021	-83.5 (-99.5, -67.4)	-89.4 (-106.1, -72.6)	-5.9 (-29.1, 17.3)
2. One-Third SD, Weighted	2018/2019	Ref	Ref	NA
	2020	-94.9 (-120, -69.7)	-99.8 (-123.3, -76.3)	-4.9 (-39.4, 29.5)
	2021	-77.3 (-100.8, -53.8)	-81 (-102.9, -59.2)	-3.7 (-35.8, 28.4)
3. Two-Thirds SD, Weighted	2018/2019	Ref	Ref	NA
	2020	-106.8 (-123.2, -90.5)	-110.8 (-127.7, -93.9)	-4 (-27.4, 19.5)
	2021	-80 (-94.5, -65.4)	-85.5 (-100.8, -70.2)	-5.5 (-26.7, 15.6)
4. One-Half SD, Unweighted	2018/2019	Ref	Ref	NA
	2020	-110.7 (-127.1, -94.3)	-118.1 (-133.5, -102.8)	-7.5 (-30, 15)
	2021	-82.9 (-97.4, -68.4)	-90 (-103.6, -76.4)	-7.1 (-27, 12.8)
**Mechanical Ventilation (Count)**				
1. One-Half SD, Weighted	2018/2019	Ref	Ref	NA
(Primary Specification)	2020	-109.2 (-124.7, -93.7)	-109.5 (-122.9, -96.1)	-0.3 (-20.8, 20.2)
	2021	-8.7 (-16.2, -1.3)	-4.7 (-14, 4.6)	4 (-7.9, 15.9)
2. One-Third SD, Weighted	2018/2019	Ref	Ref	NA
	2020	-104.5 (-127.3, -81.7)	-106.6 (-132, -81.1)	-2 (-36.2, 32.1)
	2021	-6.3 (-19.7, 7.2)	-6.7 (-19, 5.5)	-0.4 (-18.6, 17.8)
3. Two-Thirds SD, Weighted	2018/2019	Ref	Ref	NA
	2020	-103.4 (-117.4, -89.4)	-103.3 (-115.8, -90.7)	0.1 (-18.7, 18.9)
	2021	-6.8 (-13.7, 0.1)	-4.6 (-13, 3.9)	2.3 (-8.6, 13.1)
4. One-Half SD, Unweighted	2018/2019	Ref	Ref	NA
	2020	-103 (-116.8, -89.1)	-131.4 (-146.4, -116.3)	-28.4 (-48.9, -7.9)
	2021	-7.7 (-14.2, -1.2)	-11 (-20, -2)	-3.3 (-14.4, 7.8)
**Operating Margin (Point Change)**				
1. One-Half SD, Weighted	2018/2019	Ref	Ref	NA
(Primary Specification)	2020	0.2 (-0.6, 1)	1.6 (0.8, 2.4)	1.4 (0.3, 2.5)
	2021	1.5 (0.6, 2.5)	1.3 (0.3, 2.2)	-0.3 (-1.6, 1.1)
2. One-Third SD, Weighted	2018/2019	Ref	Ref	NA
	2020	0 (-1.5, 1.5)	1.5 (0.5, 2.5)	1.5 (-0.3, 3.3)
	2021	2 (0.1, 4)	1.4 (0.2, 2.7)	-0.6 (-2.9, 1.7)
3. Two-Thirds SD, Weighted	2018/2019	Ref	Ref	NA
	2020	0 (-0.7, 0.8)	1.4 (0.7, 2.1)	1.3 (0.3, 2.4)
	2021	1.7 (0.8, 2.7)	1.2 (0.3, 2.1)	-0.5 (-1.8, 0.7)
4. One-Half SD, Unweighted	2018/2019	Ref	Ref	NA
	2020	-0.1 (-0.9, 0.6)	2.1 (1.4, 2.7)	2.2 (1.2, 3.2)
	2021	1.2 (0.3, 2.2)	1.8 (1, 2.6)	0.6 (-0.6, 1.8)
**Operating Revenues (Percent Change)**				
1. One-Half SD, Weighted	2018/2019	Ref	Ref	NA
(Primary Specification)	2020	4.5 (3, 5.9)	6.1 (4.6, 7.6)	1.6 (-0.4, 3.7)
	2021	13.3 (11.4, 15.2)	11.7 (9.7, 13.7)	-1.6 (-4.3, 1.2)
2. One-Third SD, Weighted	2018/2019	Ref	Ref	NA
	2020	4.8 (1, 8.6)	5.8 (3.3, 8.3)	1 (-3.6, 5.5)

© 2025 Buxbaum JD. *JAMA Health Forum*.

	2021	13.3 (8.9, 17.7)	10.7 (8.2, 13.2)	-2.6 (-7.7, 2.5)
3. Two-Thirds SD, Weighted	2018/2019	Ref	Ref	NA
	2020	5 (3.6, 6.3)	6 (4.6, 7.5)	1.1 (-0.9, 3.1)
	2021	14 (12.3, 15.8)	12 (10.2, 13.8)	-2.1 (-4.6, 0.5)
4. One-Half SD, Unweighted	2018/2019	Ref	Ref	NA
	2020	4.6 (3, 6.2)	6.3 (5, 7.6)	1.7 (-0.3, 3.8)
	2021	13.5 (11.6, 15.3)	11.9 (10, 13.8)	-1.5 (-4.2, 1.1)
**Renal Dialysis (Count)**				
1. One-Half SD, Weighted	2018/2019	Ref	Ref	NA
(Primary Specification)	2020	-84.9 (-97.1, -72.7)	-84 (-94.8, -73.2)	0.9 (-15.4, 17.1)
	2021	-24.7 (-30.9, -18.5)	-25.5 (-32.5, -18.6)	-0.8 (-10.2, 8.5)
2. One-Third SD, Weighted	2018/2019	Ref	Ref	NA
	2020	-83.7 (-101.5, -66)	-81.8 (-97.3, -66.3)	2 (-21.6, 25.5)
	2021	-23.2 (-33.3, -13.2)	-26.1 (-33.2, -19.1)	-2.9 (-15.2, 9.4)
3. Two-Thirds SD, Weighted	2018/2019	Ref	Ref	NA
	2020	-81.6 (-92.7, -70.6)	-81.2 (-91, -71.3)	0.5 (-14.3, 15.2)
	2021	-23.9 (-29.6, -18.2)	-24.5 (-30.5, -18.6)	-0.6 (-8.9, 7.7)
4. One-Half SD, Unweighted	2018/2019	Ref	Ref	NA
	2020	-74.1 (-83.9, -64.3)	-113.9 (-127.8, -99.9)	-39.8 (-56.8, -22.7)
	2021	-21.1 (-26.2, -15.9)	-40.5 (-49.1, -31.9)	-19.4 (-29.5, -9.4)
**Total Costs - Admin (Percent Change)**				
1. One-Half SD, Weighted	2018/2019	Ref	Ref	NA
(Primary Specification)	2020	8.6 (6.6, 10.6)	8.5 (6.5, 10.5)	-0.1 (-2.9, 2.7)
	2021	13.4 (10.8, 15.9)	13.3 (10.8, 15.9)	0 (-3.7, 3.6)
2. One-Third SD, Weighted	2018/2019	Ref	Ref	NA
	2020	8.6 (5.8, 11.3)	9.1 (6.7, 11.5)	0.6 (-3.1, 4.3)
	2021	14.1 (10.2, 18)	11.4 (8.4, 14.4)	-2.7 (-7.6, 2.2)
3. Two-Thirds SD, Weighted	2018/2019	Ref	Ref	NA
	2020	8.9 (7, 10.7)	7.7 (5.6, 9.9)	-1.1 (-3.9, 1.7)
	2021	14 (11.8, 16.2)	13.4 (11.1, 15.7)	-0.6 (-3.8, 2.6)
4. One-Half SD, Unweighted	2018/2019	Ref	Ref	NA
	2020	9 (7, 10.9)	8.8 (7.1, 10.6)	-0.1 (-2.8, 2.5)
	2021	13.2 (10.8, 15.5)	12.4 (10.4, 14.4)	-0.8 (-3.9, 2.3)
**Total Costs - All (Percent Change)**				
1. One-Half SD, Weighted	2018/2019	Ref	Ref	NA
(Primary Specification)	2020	4.5 (3.4, 5.7)	4.6 (3.6, 5.6)	0 (-1.5, 1.6)
	2021	11.8 (10.1, 13.6)	11.6 (10.2, 13.1)	-0.2 (-2.5, 2.1)
2. One-Third SD, Weighted	2018/2019	Ref	Ref	NA
	2020	4.4 (2.2, 6.6)	3.5 (2, 5)	-0.9 (-3.5, 1.8)
	2021	10.5 (7.2, 13.9)	9.7 (7.6, 11.7)	-0.9 (-4.8, 3.1)
3. Two-Thirds SD, Weighted	2018/2019	Ref	Ref	NA
	2020	4.9 (3.7, 6)	4.5 (3.5, 5.4)	-0.4 (-1.9, 1.1)
	2021	12.1 (10.5, 13.7)	11.4 (10.1, 12.8)	-0.7 (-2.7, 1.4)
4. One-Half SD, Unweighted	2018/2019	Ref	Ref	NA
	2020	5 (3.9, 6)	4.6 (3.7, 5.4)	-0.4 (-1.7, 1)
	2021	12.2 (10.7, 13.6)	11.3 (10.1, 12.5)	-0.9 (-2.7, 1)
**Total Costs - Workforce (Percent Change)**				
1. One-Half SD, Weighted	2018/2019	Ref	Ref	NA
(Primary Specification)	2020	3.2 (1.8, 4.7)	3.1 (1.8, 4.3)	-0.2 (-2.1, 1.7)
	2021	10.8 (8.5, 13.2)	11.2 (9.2, 13.1)	0.3 (-2.7, 3.3)
2. One-Third SD, Weighted	2018/2019	Ref	Ref	NA
	2020	3.7 (1.4, 6)	2.2 (0.5, 3.9)	-1.5 (-4.3, 1.4)
	2021	9.7 (5.4, 14.1)	9.5 (7.1, 12)	-0.2 (-5.2, 4.8)

© 2025 Buxbaum JD. *JAMA Health Forum*.

3. Two-Thirds SD, Weighted	2018/2019	Ref	Ref	NA
	2020	3.5 (2.1, 4.8)	2.7 (1.5, 3.9)	-0.8 (-2.6, 1)
	2021	11.5 (9.4, 13.6)	10.8 (9.1, 12.6)	-0.7 (-3.4, 2.1)
4. One-Half SD, Unweighted	2018/2019	Ref	Ref	NA
	2020	3.8 (2.5, 5)	3.2 (2.3, 4.1)	-0.5 (-2.1, 1)
	2021	11.9 (10, 13.7)	11.1 (9.6, 12.5)	-0.8 (-3.2, 1.6)

© 2025 Buxbaum JD. *JAMA Health Forum*.
