# Supplementary material for: Enhanced COVID-19 Provider Relief, Hospital Finances, and Care for Medicare Inpatients
Source: JAMA Health Forum. 2025 Mar 7;6(3):e250046. doi: 10.1001/jamahealthforum.2025.0046 (PMC11889469; doi:10.1001/jamahealthforum.2025.0046)
Supplement: Supplement 2. — Data Sharing Statement [file jamahealthforum-e250046-s002.pdf]

Data Sharing Statement

Buxbaum. Enhanced COVID-19 Provider Relief, Hospital Finances, and Care for Medicare Inpatients. *JAMA Health Forum*. Published March 07, 2025.
doi:10.1001/jamahealthforum.2025.0046

Data
Data available: Yes
Data types: Data (not involving human participants), Data dictionary, Other (please specify)
Additional Information: Case counts used to determine distribution of high-impact funds
How to access data: Data available at https://github.com/jbuxbaum.
When available: With publication

Supporting Documents
Document types: Statistical/analytic code
How to access documents: Data available at https://github.com/jbuxbaum.
When available: With publication

Additional Information
Who can access the data: All
Types of analyses: All
Mechanisms of data availability: NA
Any additional restrictions: Request for citation to accompanying JAMA Health Forum article.
